# Computed tomography features of spontaneous acute intracranial hemorrhages in a tertiary hospital in Southern Ghana

**DOI:** 10.11604/pamj.2021.40.226.31934

**Published:** 2021-12-16

**Authors:** Emmanuel Kobina Mesi Edzie, Klenam Dzefi-Tettey, Edmund Kwakye Brakohiapa, Philip Narteh Gorleku, Eric Aidoo, Kwasi Agyen-Mensah, Peter Appiah-Thompson, Adu Tutu Amankwa, Ewurama Andam Idun, Frank Quarshie, Richard Ato Edzie, Benard Osei, Prosper Dziwornu, Abdul Raman Asemah

**Affiliations:** 1 Department of Medical Imaging, School of Medical Sciences, College of Health and Allied Sciences, University of Cape Coast, Cape Coast, Ghana,; 2 Department of Radiology, Cape Coast Teaching Hospital, Cape Coast, Ghana,; 3 Department of Radiology, Korle Bu Teaching Hospital, 1 Guggisberg Avenue, Accra, Ghana,; 4 Department of Radiology, University of Ghana Medical School, Accra, Ghana,; 5 Department of Anatomy, School of Medical Sciences, College of Health and Allied Sciences, University of Cape Coast, Cape Coast, Ghana,; 6 Department of Neurosurgery, School of Medical Sciences, College of Health and Allied Sciences, University of Cape Coast, Cape Coast, Ghana,; 7 Department of Surgery, School of Medical Sciences, College of Health and Allied Sciences, University of Cape Coast, Cape Coast, Ghana,; 8 Department of Radiology, School of Medical Sciences, College of Health Sciences, Kwame Nkrumah University of Science and Technology, Kumasi, Ghana,; 9 Department of Radiology, 37 Military Hospital, Neghelli Barracks Liberation Road 37, Accra, Ghana,; 10 African Institute for Mathematical Sciences (AIMS), Summerhill Estates, East Legon Hills, Santoe, Accra, Ghana

**Keywords:** Computed tomography, features, spontaneous acute intracranial hemorrhages, Ghana

## Abstract

**Introduction:**

spontaneous acute intracranial hemorrhage (SICH) accounts for approximately 10-15% of all stroke cases. Early detection by computed tomography (CT) and early treatment are key. Hence this study to examine the CT features of SICH.

**Methods:**

this retrospective cohort study reviewed all 435 patients diagnosed with SICH from 1^st^ March, 2017 to 1^st^ January, 2021 in a tertiary facility in Southern Ghana. Data collected (age, sex, SICH type and the CT scan features) were organized and analyzed using GNU PSPP and Libre Office Calc. Statistical significance level was pegged at p≤0.05.

**Results:**

the SICH types were acute intracerebral hemorrhage (97.93%), acute subarachnoid/intraventricular hemorrhage (1.15%), acute epidural hemorrhage (0.46%) and acute subdural hemorrhage (0.46%). Acute intracerebral hemorrhage was more common in those >60 years (57.75%, p<0.001). The commonest CT feature for acute intracerebral hemorrhage was hyperdense lesion with perilesional edema (40.98%), with smoking (OR=2.24, 95% CI: 1.14-4.41, p=0.019) and anticoagulants intake (OR=2.56, 95% CI: 1.15-5.72, p=0.022) as the predictive factors; followed by hyperdense lesion extending to the edge of the brain (25.03%), also predictable by smoking (OR=0.23, 95% CI: 0.11-0.47, p<0.001); and hyperdense lesion with mass effects (22.70%) was not predictive with any risk factor. Type 2 diabetes mellitus (60.00%, p<0.001) and smoking (97.83%, p<0.001) were more common in males.

**Conclusion:**

hyperdense lesion with perilesional edema was the most frequent CT feature for acute intracerebral hemorrhage and was predictable by smoking and anticoagulants intake. Smoking was a predictive factor to the development of most of the features of acute intracerebral hemorrhage.

## Introduction

Spontaneous intracranial hemorrhage (SICH) is an important neurologic condition that occurs due to non-traumatic bleeding inside the skull, leading to significant morbidity and high fatality rate [1,2]. Intracranial hemorrhage accounts for approximately 10-15% of all stroke cases, and SICH, vascular malformations, ruptured berry aneurysm, and bleeding associated with bleeding disorders are largely responsible [3,4]. The incidence and prevalence of SICH cases are now increasing in the low- and middle-income countries and are much higher than in western countries [5-8]. The anatomical location of hemorrhage is crucial in differentiating the type of SICH, thus may occur as intracerebral hemorrhage, subarachnoid hemorrhage, or intraventricular hemorrhage, subdural hemorrhage and epidural hemorrhage [9]. Risk factors for spontaneous intracranial hemorrhage include old age, cigarette smoking, alcohol use, hypertension, cocaine addiction and patients treated with anticoagulants. Common clinical features include headache, nausea, vomiting, confusion, somnolence, or seizure [10]. Early detection of the etiology of bleeding is key to the management of spontaneous intracranial hemorrhage [11]. Before the advent of computed tomography (CT), diagnosis was based on clinical experience, cerebral angiography, plain skull roentgenograms, and isotope brain scan. However, the introduction of CT in 1972, has significantly improved the accuracy and diagnosis of intracranial hemorrhage, and has become the neuro-imaging modality of choice in the assessment of SICH [2,12,13]. Even though CT scan has been reported in a recent study in Ghana as one of the most available imaging modalities in radiological practices and hence the most employed modality for neuroimaging, there are some challenges like relatively high cost, frequent power failures and few others which may end up delaying early detection of SICH [14-16]. Therefore, we aimed at analyzing the CT scan patterns in patients with spontaneous intracranial hemorrhage in a tertiary care hospital located in the Central Region of Ghana. The specific objectives of the study were to: i) determine the proportions of the various types of acute intracranial hemorrhage; ii) determine any possible association between age, sex, and intracranial hemorrhage; iii) ascertain the commonest features of acute intracranial hemorrhage in the Ghanaian setting, and their predictive risk factors.

## Methods

**Study design, site and participants:** this was a hospital-based retrospective cohort study conducted at the Cape Coast Teaching Hospital (CCTH), which retrieved all non-contrast head CT scans for spontaneous acute intracranial hemorrhage (intracerebral, subarachnoid/intraventricular, subdural, and epidural hemorrhages) from 1^st^ March, 2017 to 1^st^ January, 2021. The CCTH is the only tertiary public health facility which serves as a referral center for the inhabitants of the Central Region of Ghana and its environs. The facility is equipped with modern imaging modalities with the exception of magnetic resonance imaging (MRI). The CCTH is situated in the coastal city of Cape Coast, the Central Regional capital; it is a 400-bed capacity facility and currently the professional training center for clinical internships of the schools of nursing, medical and allied health sciences of the University of Cape Coast in the South-Central part of Ghana. A total of 435 CT scan diagnosed spontaneous acute intracranial hemorrhages were consecutively retrieved and evaluated for analysis.

**Computed tomography scan image acquisition and interpretation:** computed tomography scanner, model TSX-101A manufactured by Toshiba medical systems (Otawara, Tochigi, Japan), which is a 16 slice multi-detector machine was used in the acquisition of the head CT scans from the base to the vertex of the skull. The CT scan parameters employed were; slice thickness of 5mm, tube current-exposure time of 225mAs, collimation 1x16, tube voltage 120 kV and rotation time 0.75s. The images were then transferred and stored in the Picture Archiving Communication System (PACS) (IBM Watson Health Global Headquarters, Cambridge, MA, USA). The PACS images for SICH were consecutively retrieved and reviewed by one radiographer and 3 independent radiologists with over 8 years of experience in performing and reporting head CT scans without any exclusions. Radiological features considered for intracranial hemorrhages included intra-cerebral and/or extra-cerebral hyperdense lesions with acute blood attenuation, perilesional edema, mass effect depending on the extent of lesion, with/without intraventricular extension. For acute intracerebral hemorrhages, the following features were considered: hyperdense lesion (white area) extending to the edge of the brain, involving both the gray and white matter; hyperdense lesion with perilesional hypodensity/edema; hyperdense lesion with mass effects (compression of the adjacent structures)/mid-line shift to the opposite side; hyperdense lesion with extension into ventricular systems; and small hyperdensities deep in the brain/petechiae hemorrhages [17].

For acute subdural hemorrhages, we considered the following features: hyperdense (white/bright) crescentic lesion between the brain and the skull; lesion crosses suture lines but never crosses dural reflections (falx cerebri and the tentorium); mass effects/mid-line shift to the opposite side; loss of gyri and sulci prominence. Acute epidural hemorrhage features were: hyperdense (white/bright) biconvex lens-shaped lesion between the brain and the skull; lesion does not cross suture lines but may cross dural reflections (falx cerebri and the tentorium); hyperdense lesion with mass effects/mid-line shift to the opposite side; loss of gyri and sulci prominence [17]. Subarachnoid/intraventricular hemorrhages were diagnosed with the following features: hyperdense cerebrospinal fluid (CSF) instead of its usual hypodense appearance; hyperdensities in the external CSF spaces (Sylvian fissure, the suprasellar cistern, the basal cistern and the quadrigeminal cistern); intraventricular hyperdensities especially in the occipital horns of the lateral ventricles. Disagreement between reports were settled by mutual and collective engagement to build consensus [17].

**Data collection:** the age, sex, CT scan features for SICH, and risk factors (including dyslipidemia, type 2 diabetes mellitus (DM-2), hypertension, smoking and patients on anti-coagulants), were also obtained from the lightwave health management information system (LHMIS) of the CCTH by the investigators. Tobacco smoking meant any person who has smoked 100 or more sticks of cigarettes in their lifetime [18]. Dyslipidemia was defined as people with cholesterol lipoprotein ≥140mg/dL, triglycerides > 200 mg/dL, high density lipoprotein < 40mg/dL and/or total cholesterol >200mg/dL. Type 2 diabetes mellitus (DM-2) in our study means a person with blood sugar level ≥ 200mg/dL [19]. Hypertension meant a sustained elevation of blood pressure (BP) > 140/90mmHg in line with the International Society of Hypertension and World Health Organization (WHO) guidelines [20]. The age was also categorized as “<20 years,” “20-40 years,” “41-60 years,” and “>60 years,” to help determine the distribution of SICH types and risk factors among the age groups.

**Statistical analysis:** the data obtained were organized, inputted for analyses using GNU PSPP (pspp version 1.2.0-3, category: education, science and math, developed by the free software foundation), to obtain percentages, tables and frequencies. The charts were obtained using the LibreOffice Calc (version 1: 6.1.5-3+deb10u6, developed by the Document Foundation). Chi-squared test of independence was used to test for association between variables (risk factors against age and sex, and also intracranial hemorrhage types with age, sex). We predicted the development of the commonest CT scan features with the risk factors, using logistic regression analysis, after the goodness of fit test criteria of the models had been met. Statistical significance was set at p≤0.05.

**Exclusion criteria:** post-traumatic intracranial hemorrhages were excluded.

**Ethical consideration:** ethical clearance number (CCTHERC/EC/2020/092) for this study was obtained from the Ethical Review Committee of CCTH. Informed consent was not required for this study as this was a retrospective study but anonymity and confidentiality were ensured. This study conformed to the 1975 Declaration of Helsinki.

## Results

**Figure 1:**
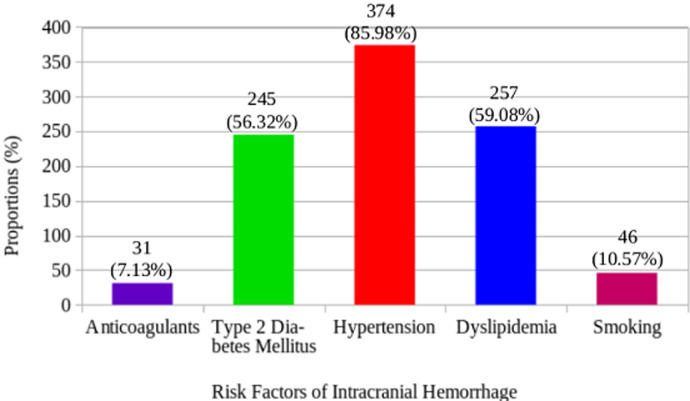
risk factors of intracranial hemorrhage

Out of the 435 participants included in the study, 51.49% were males and 48.51% were females. The mean age of the study population was 61.25 (SD=17.25) years, ranging from 15-100 years. Only two of the participants were below the age of 20 years. The majority of them were more than 60 years (56.78%), followed by those who were 41-60 years and 20-40 years constituting 28.74% and 14.02% respectively as shown in Table 1. The most common risk factor in our study was hypertension (85.98%), followed by dyslipidemia (59.08%) and type 2 diabetes mellitus (56.32%). Only 7.13% and 10.57% of the participants had history of taking anticoagulants and smoking respectively, as shown in Figure 1. None of the participants below 20 years had dyslipidemia or had a history of smoking or taking anticoagulants. The risk factors considered like anticoagulants intake, smoking, type 2 diabetes mellitus (DM-2), dyslipidemia and hypertension were all common in those more than 60 years, constituting 48.39%, 50.00%, 55.92%, 55.25%, and 56.15% respectively, but DM-2 increased significantly with age (p=0.007). 52.41% of the participants with hypertension were males and 47.59% were females, with p=0.347. Most of the participants with dyslipidemia were females (51.36%) and the remaining (48.64%) were males but statistically, there was no difference between them (p=0.152). A major proportion of those who were taking anticoagulants were also males (67.74%). The other results on the association between the risk factors and socio-demographics (age and sex) are shown in Table 2.

**Table 1:** socio-demographics of participants

Variable	Count/frequency	Percentage (%)
**Age**		
Minimum	15 years	
Maximum	100 years	
Mean (SD)	61.25 (17.25) years	
**Age group**		
<20 years	2	0.46%
20-40 years	61	14.02%
41-60 years	125	28.74%
>60 years	247	56.78%
**Sex**		
Males	224	51.49%
Females	211	48.51%

**Table 2:** distribution of the risk factors among age and sex

Variable	Age group				P-value
Risk factors	<20years	20-40 years	41-60years	>60years	
Anticoagulants	-	5(16.13%)	11(35.48%)	15(48.39%)	0.752
Smoking	-	3(6.52%)	20(42.48%)	23(50.00%)	0.086
DM-2	2(0.82%)	45(18.37%)	61(24.90%)	137(55.92%)	0.007*
Dyslipidemia	-	33(12.84%)	82(31.91%)	142(55.25%)	0.113
Hypertension	2(0.53%)	48(12.83%)	114(30.48%)	210(56.15%)	0.110
	**Sex**				
	**Males**		**Females**		
Anticoagulants	21(67.74%)		10(32.26%)		0.060
Smoking	45(97.83%)		1(2.17%)		<0.001*
DM-2	147(60.00%)		98(40.00%)		<0.001*
Dyslipidemia	125(48.64%)		132(51.36%)		0.152
Hypertension	196(52.41%)		178(47.59%)		0.346

* Statistically significant

**Figure 2:**
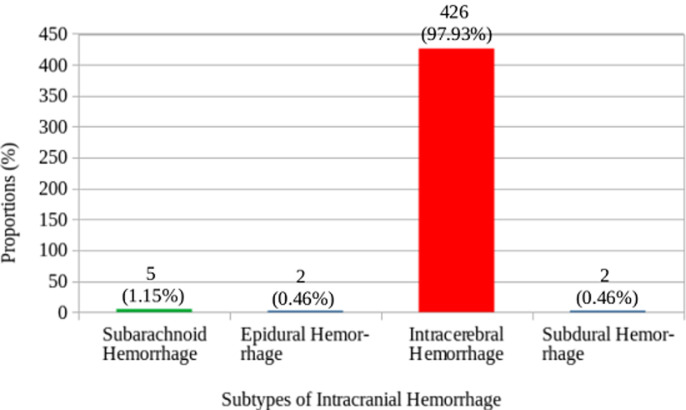
proportions of the subtypes of intracranial hemorrhage

**Figure 3:**
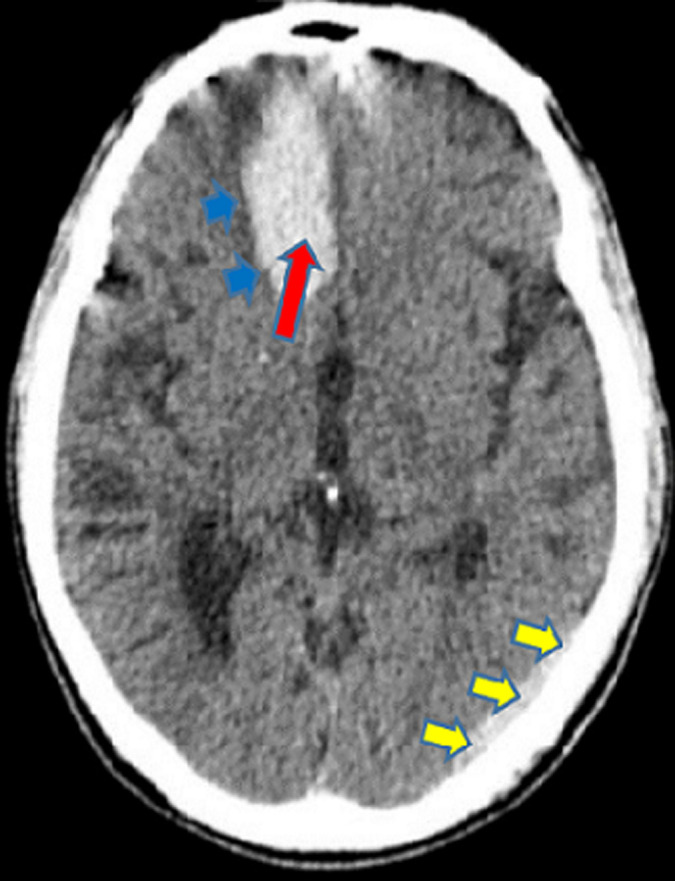
an axial non-enhanced CT scan of the head showing an acute right frontal intraparenchymal hemorrhage (red arrow), perilesional edema without a midline shift (blue short arrows), and subtle acute left occipital epidural hematoma (yellow short arrows)

**Figure 4:**
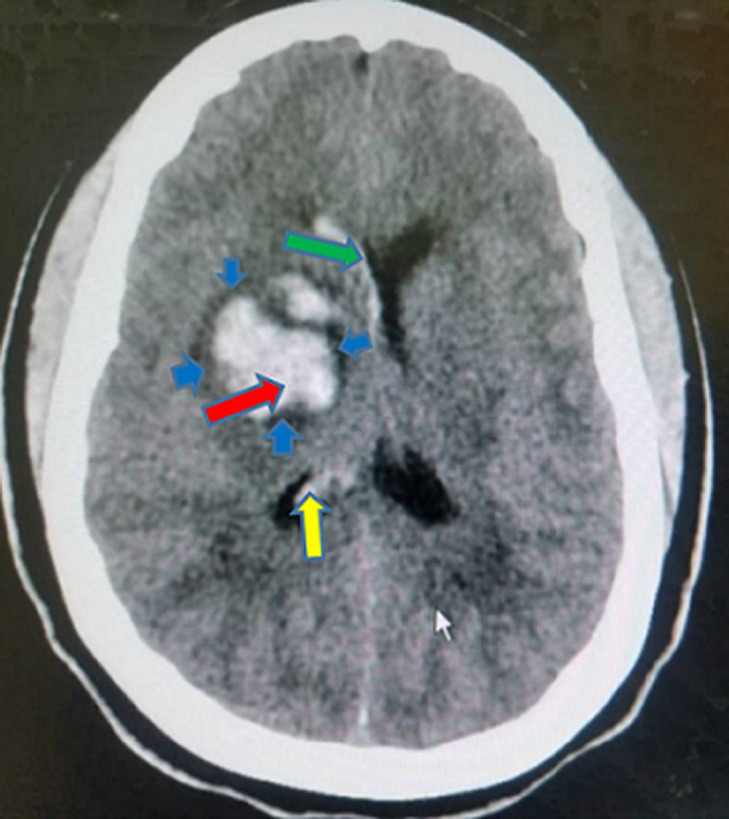
a non-contrast axial CT scan of the head showing an acute right basal ganglia hemorrhage (red arrow), perilesional edema (blue short arrows), extension into the ipsilateral lateral ventricle (yellow arrow) and mass effect (green arrow)

**Figure 5:**
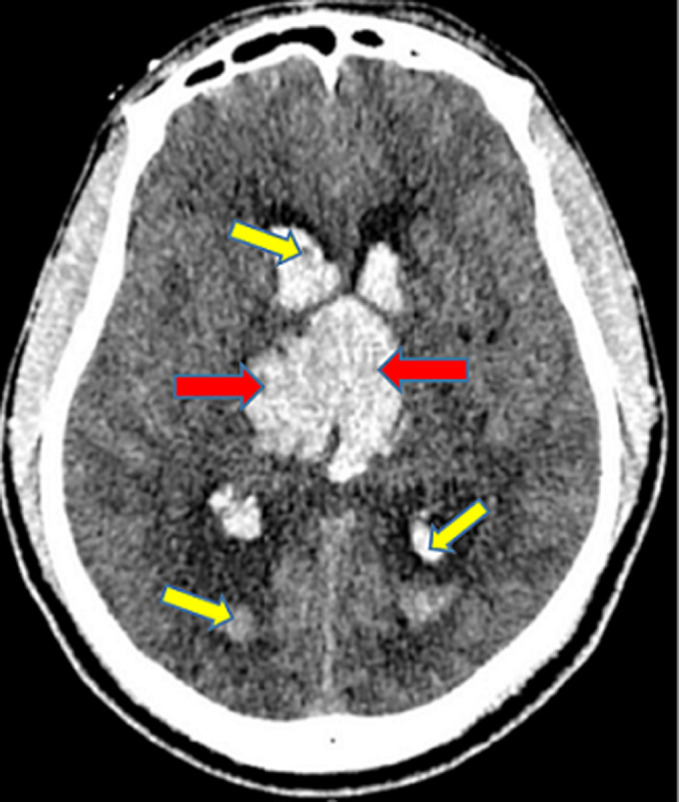
an axial non-enhanced CT scan of the brain showing hyperdense collections of acute bleed in both thalami (red arrows) and extension into the lateral ventricles (yellow arrows)

**Figure 6:**
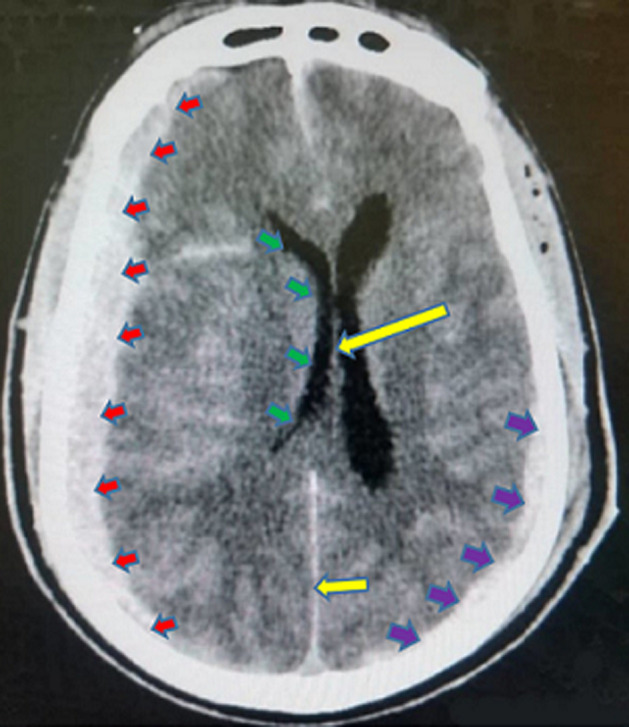
a non-contrast head CT scan showing acute subdural hematomas with loss of gyri and sulci (red short and violet arrows), mass effect (green short arrows), midline shift (yellow arrow) compared to the normal midline position (yellow short arrow)

In Figure 2 shows the proportions of the various types of intracranial hemorrhage seen in this study. The huge majority of the participants in the study suffered intracerebral hemorrhage (97.93%), followed by subarachnoid/intraventricular hemorrhage (1.15%). Those who had spontaneous epidural and subdural hemorrhages constituted 0.46% each. Out of the total number of the participants who had intracerebral hemorrhage, 51.41% were males and 48.59% were females. All the two participants who had epidural hemorrhage were males. Exactly half (50.00%) of those who suffered subdural hemorrhage and 60.00% of those who had subarachnoid/intraventricular hemorrhage were females. The intracerebral hemorrhage was significantly more common in those who were more than 60 years (57.75%, p<0.001) and the subarachnoid/intraventricular hemorrhage was more common in those who were 20-40 years (60.00%, p<0.001). The rest are shown in Table 3. The commonest CT scan feature for the intracerebral hemorrhage was hyperdense lesion with perilesional hypodensity/edema (40.98%), followed by hyperdense lesion extending to the edge of the brain (25.03%) (Figure 3) and hyperdense lesion with mass effects/mid-line shift to the opposite side (22.70%) (Figure 4). The next commonest feature was hyperdense lesion with extension into ventricular systems (9.69%) (Figure 4, Figure 5). The CT scan features for subdural hemorrhage were hyperdense crescentic lesion with mass effects/mid-line shift to the opposite side; hyperdense crescentic lesion between the brain and the skull; crosses suture lines but never crosses dural reflections; and loss of gyri and sulci prominence (Figure 6). All these features showed equal distribution thus 25.00% each (Table 4).

**Table 3:** distribution of the intracranial hemorrhage types among sex and age group

Sex	Intracerebral hemorrhage	Epidural hemorrhage	Subdural hemorrhage	Subarachnoid hemorrhage	P-value
Males	219 (51.41%)	2 (100.00%)	1 (50.00%)	2 (40.00%)	0.542
Females	207 (48.59%)	-	1 (50.00%)	3 (60.00%)	
**Age group**					
<20 years	1 (0.23%)	-	1 (50.00%)	-	<0.001*
20-40 years	57 (13.38%)	-	1 (50.00%)	3 (60.00%)	
41-60 years	122 (28.64%)	1 (50.00%)	-	2 (40.00%	
>60 years	246 (57.75%)	1 (50.00%)	-	-	

*Statistically significant

**Table 4:** proportions of CT scan features/patterns for intracerebral and subdural hemorrhages

Proportions of CT scan features for intracerebral hemorrhage (n=426)		
	Count	Percentage
Hyperdense lesion extending to the edge of the brain	204	25.03%
Hyperdense lesion with perilesional hypodensity/edema	334	40.98%
Hyperdense lesion with mass effects/mid-line shift to the opposite side	185	22.70%
Hyperdense lesion with extension into ventricular systems	79	9.69%
Small hyperdensity deep in the brain/petechiae	13	1.60%
**Total**	815	100.00%
**Proportions of CT scan features for subdural hemorrhage (n=2)**		
Hyperdense crescentic lesion between the brain and the skull	2	25.00%
Crosses suture lines but never crosses dural reflections	2	25.00%
Hyperdense crescentic lesion with mass effects/mid-line shift to the opposite side	2	25.00%
Loss of gyri and sulci prominence	2	25.00%
**Total**	8	100.00%

**Figure 7:**
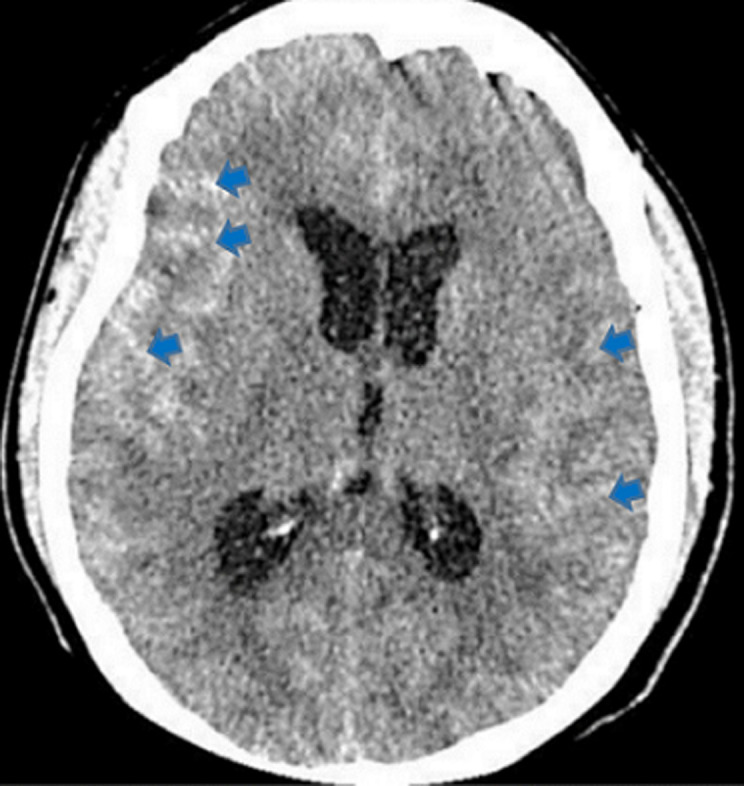
an axial non-enhanced CT scan of the head showing hyperdense collections of acute blood attenuation in the sulci in keeping with acute subarachnoid hemorrhage (blue short arrows)

The CT scan features of acute epidural hemorrhage were hyperdense biconvex lens-shaped lesion between the brain and skull (25.00%); mass effects/mid-line shift to the opposite side (25.00%); does not cross suture lines but may cross dural reflections (25.00%); and loss of gyri and sulci prominence (25.00%) (Figure 3). Subarachnoid/intraventricular hemorrhage in our study showed on CT scan as: hyperdense cerebrospinal fluid instead of its usual hypodense appearance (33.33%); hyperdensities in the external cerebrospinal fluid spaces (33.33%); and intraventricular hyperdensities especially in the occipital horns of the lateral ventricles (33.33%) (Figure 7), as shown in Table 5. Smoking significantly increased the chances of developing hyperdense lesion extending to the edge of the brain by 0.23-folds (95% CI: 0.11-0.47, p<0.001), hyperdense lesion with perilesional hypodensity/edema by 2.24-folds (95% CI: 1.14-4.41, p=0.019), and hyperdense lesion with extension into ventricular systems was increased by 13.22-folds (95% CI: 1.76-99.24, p=0.012). Anticoagulants intake also had significant contribution to predicting the development of hyperdense lesion with perilesional hypodensity/edema (OR=2.56, 95% CI: 0.11-0.47, p=0.022). The others are shown in Table 6.

**Table 5:** proportions of CT scan features/patterns for epidural and subarachnoid hemorrhages

Proportions of CT scan features for epidural hemorrhage (n=2)		
	Count	Percentage
Hyperdense biconvex lens-shaped lesion between the brain and skull	2	25.00%
Does not cross suture lines but may cross dural reflections	2	25.00%
Hyperdense lesion with mass effects/mid-line shift to the opposite side	2	25.00%
Loss of gyri and sulci prominence	2	25.00%
**Total**	8	100.00%
**Proportions of CT scan features for subarachnoid hemorrhage (n=5)**		
Hyperdense cerebrospinal fluid instead of its usual hypodense appearance	5	33.33%
Hyperdensities in the external cerebrospinal fluid spaces	5	33.33%
Intraventricular hyperdensities especially in the occipital Horns of the lateral ventricles	5	33.33%
**Total**	15	100.00%

**Table 6:** logistic regression analysis

Risk factors	Odd ratio (OR)	95% confidence interval (CI)	P-Value
**Hyperdense lesion extending to the edge of the brain**			
Anticoagulants	0.88	0.41-1.89	0.739
Smoking	0.23	0.11-0.47	<0.001*
Type 2 diabetes mellitus	1.25	0.79-1.99	0.339.
Dyslipidemia	0.90	0.56-1.43	0.649
Hypertension	0.80	0.45-1.41	0.438
**Hyperdense lesion with perilesional hypodensity/edema**			
Anticoagulants	2.56	1.15-5.72	0.022*
Smoking	2.24	1.14-4.41	0.019*
Type 2 diabetes mellitus	0.77	0.45-1.33	0.352
Dyslipidemia	0.88	0.51-1.50	0.628
Hypertension	1.86	0.86-4.02	0.112
**Hyperdense Lesion with mass effects/mid-line shift to the opposite side**			
Anticoagulants	1.38	0.63-3.03	0.419
Smoking	1.49	0.78-2.86	0.230
Type 2 diabetes mellitus	0.84	0.53-1.33	0.454
Dyslipidemia	1.34	0.85-2.13	0.206
Hypertension	0.80	0.45-1.42	0.450
**Hyperdense lesion with extension into ventricular systems**			
Anticoagulants	1.91	0.55-6.68	0.309
Smoking	13.22	1.76-99.24	0.012*
Type 2 diabetes mellitus	0.77	0.42-1.40	0.386
Dyslipidemia	1.87	1.02-3.42	0.042*
Hypertension	0.94	0.46-1.90	0.854

*Statistically significant

## Discussion

Aging has been reported as an important factor that contributes to increase in intracranial hemorrhages [21,22]. In our study, we found that the incidence of intracranial hemorrhage increased with age, since more than half of the participants in our study were more than 60 years (Table 1). Some studies have reported SICH being more common in females than males [9,23]. Contrary to this, our study found that, most of the participants were males (51.49%). This could be due to the inability to adhere to healthy diet and lack of exercise, thereby increasing the risk factors of SICH in men, as also corroborated by Galati *et al*. [24]. Subarachnoid/intraventricular hemorrhage was the most common type of intracranial hemorrhage as reported by Bonatti *et al*. In their study, it constituted the majority of 59.48%, followed by intracerebral hemorrhage (28.45%) and subdural hemorrhage (12.07%). The authors recorded no cases of epidural hemorrhage [9]. Their findings are contrary to ours, as the majority of the participants presented with intracerebral hemorrhage (97.93%), followed by subarachnoid/intraventricular hemorrhage (1.15%) but the occurrence of subdural and epidural hemorrhages were the same (0.46% each). In Ghana, there are very few reported studies on spontaneous intracranial hemorrhage, Obajimi *et al*. also reported intracerebral hemorrhage as the most common type of SICH in agreement with what we found [25]. The intracerebral hemorrhage found in this current study was significantly associated with increasing age, which has also been corroborated by Forti *et al*. in Italy [26]. Hypertension was the most common risk factor for SICH in our study, which affected more than 85% of the participants, followed by dyslipidemia (59.08%) and DM-2 (56.32%). This was similar to what was reported by Neuberger *et al*. [27].In their study, they recorded 69.8% of the patients presenting with hypertension, 28.4% with dylipidemia and 22.22% with DM-2, but we had comparatively higher proportions (Figure 1). Diabetes mellitus-2 and smoking were found to be significantly associated with sex with p-value <0.001 (affecting more males). This could be the reason why the SICH was more common in males in our study. However, DM-2 was significantly more common in the aged (>60 years), (p=0.007).

In our study, hyperdense lesion with perilesional hypodensity/edema was the commonest (40.98%) CT scan feature for intracerebral hemorrhage, followed by hyperdense lesion extending to the edge of the brain (25.03%) (Figure 3). Some studies have reported these CT scan features as the most frequent [17,28]. Vilela *et al*. in another study, reported that hyperdense lesions on CT scan constituted 40-60% of all acute intracerebral hemorrhages, which purports that it is the most prevalent feature as also seen in this study [2]. The CT scan features for subdural, epidural and subarachnoid/intraventricular hemorrhages in our study had same proportions (Table 4, Table 5). Our study found that, smoking as a risk factor, could predict the occurrence of the commonest CT scan feature (OR=2.24, 95% CI: 1.14-4.41, p=0.019); the second-commonest CT scan feature (OR=0.23, 95% CI: 0.11-0.47, p<0.001); and the fourth-commonest CT scan feature (OR=13.22, 95% CI: 1.76-99.24, p=0.012). Hyperdense lesion with mass effects (compression of the adjacent structures)/mid-line shift to the opposite side (Figure 4), the third-commonest CT scan feature, was not predictable by any of the risk factors in the study and hyperdense lesion with extension into ventricular systems (Figure 5), was also predictable by dyslipidemia (Table 6). Reduction of these risk factors could decrease the occurrence of these CT scan features of spontaneous acute intracranial hemorrhage [29,30]. Strokes are generally known to be a significant cause of morbidity and mortality in the world and Africa in particular. Its incidence has been reported to be increasing with increasing age and as the years go by [16,21]. Therefore, the findings from this current study emphasize the essence of knowing the radiological features of CT scan for SICH in order to enable quick and accurate diagnosis, as the management of such events depends on how early the diagnosis is made. We therefore recommend that practitioners should also be aware of the CT scan features of SICH for quick identification, diagnosis and management.

## Conclusion

Acute intracerebral hemorrhage was the most common type of spontaneous acute intracranial hemorrhage, followed by subarachnoid/intraventricular hemorrhage. Hyperdense lesion with perilesional hypodensity due to edema was the commonest CT scan feature for spontaneous acute intracerebral hemorrhage, which was predictable by smoking and intake of anticoagulants. Smoking was a predictive risk factor to most of the CT scan features. Even though spontaneous acute intracranial hemorrhages from our study increased with increasing age, we realized the acute subarachnoid hemorrhage was more common in the younger age group.

### What is known about this topic

- Spontaneous acute intracranial hemorrhage presents as hyperdense intracerebral area with fresh blood attenuation on non-contrast CT scan images.

### What this study adds

- This study reports on which risk factor can predict the development of the commonest CT scan features of spontaneous acute intracranial hemorrhage, and was found that the occurrence of most of the CT scan features of spontaneous acute intracranial hemorrhage can be predicted by smoking.
